# Axial Round Cell Sarcoma Harboring a Non-ETS EWSR1 Rearrangement: Diagnostic Challenges and Clinical Implications

**DOI:** 10.7759/cureus.103821

**Published:** 2026-02-18

**Authors:** Sergio Bolivar, Leidy P Cespedes Useche, Oscar I Reyes, Jorge Aponte

**Affiliations:** 1 Internal Medicine, Universidad Militar Nueva Granada, Hospital Militar Central, Bogotá, COL; 2 Hematology and Medical Oncology, Hospital Militar Central, Bogotá, COL; 3 Oncology, Clinica Colombia, Bogotá, COL

**Keywords:** adult soft tissue sarcoma, bcor rearranged sarcoma, ewsr1-rearranged sarcoma, non-ets fusion, spindle and round cell sarcoma

## Abstract

We present a rare case of a cervicothoracic epidural spindle and round cell sarcoma in a 59-year-old man, characterized by an EWSR1 gene rearrangement. The patient experienced progressive cervical pain and lower limb weakness due to an extradural mass at the C7-T2 level. Surgical resection and cervicothoracic fixation were performed, followed by radiotherapy (30 Gy/10 fractions) and Ewing-based chemotherapy (doxorubicin/ifosfamide). Histopathological analysis revealed a spindle and oval cell neoplasm with a Ki-67 index of 30%. The tumor was positive for CD99, SATB2, TLE1, cyclin D1, and focal FLI1, while negative for EMA, S100, desmin, calponin, and SOX10. Fluorescence in situ hybridization (FISH) analysis confirmed EWSR1 break-apart signals (3-8) in 70% of nuclei and separation in 18% of cells, indicating an EWSR1-non-ETS fusion. Local recurrence occurred despite multimodal therapy. This case highlights the clinical and diagnostic challenges associated with EWSR1-rearranged non-ETS sarcomas, which exhibit distinct molecular behaviors, morphology, and treatment responses compared to classical Ewing sarcoma.

## Introduction

EWSR1-rearranged sarcomas comprise a genetically heterogeneous group of neoplasms that include conventional Ewing sarcoma and several non-ETS fusion variants [1,2]. Classical Ewing sarcoma is defined by EWSR1-FLI1 or EWSR1-ERG fusions involving members of the ETS transcription factor family [1]. In contrast, non-ETS fusions such as EWSR1-NFATC2, EWSR1-CREB1, and EWSR1-ATF1 delineate distinct molecular subtypes with unique morphologic features and clinical behavior [3-5]. Primary epidural or paraspinal EWSR1-rearranged sarcomas are exceptionally rare in adults [6]. The rarity of non-ETS fusion EWSR1-rearranged sarcomas in adults necessitates heightened awareness among clinicians to ensure timely diagnosis and appropriate management strategies. These tumors often exhibit unique histopathological features and may not respond effectively to conventional Ewing sarcoma therapies, highlighting the need for tailored treatment approaches. In conclusion, recognizing the distinct characteristics of EWSR1-rearranged sarcomas is crucial for accurate diagnosis and effective treatment, as these tumors may exhibit resistance to standard Ewing sarcoma therapies.

## Case presentation

**Figure 1:**
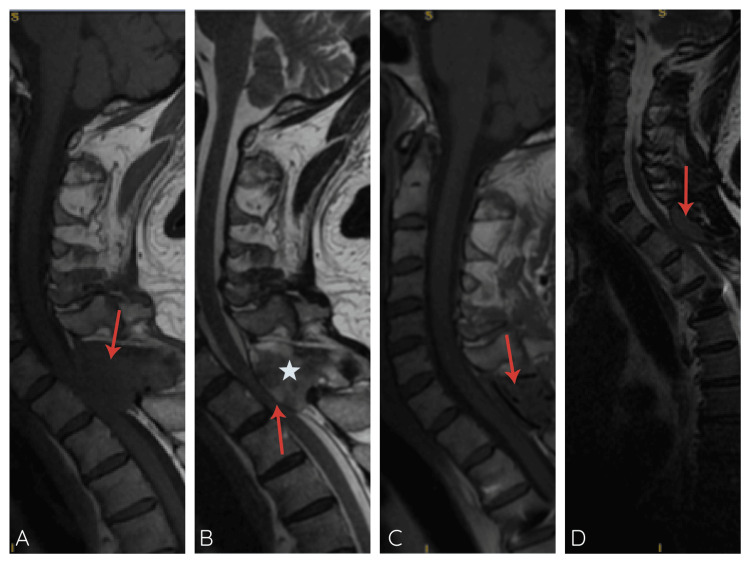
Magnetic resonance imaging (MRI) of the cervical spine (A) T1-weighted MRI sequence showing a lesion with signal intensity like that of adjacent soft tissues (red arrow). (B) T2-weighted MRI sequence revealing a heterogeneous mass involving the posterior paraspinal tissues (star), with contiguous epidural extension and spinal cord compression (red arrow). (C) Post-chemotherapy T1-weighted sequence, demonstrating a partial treatment response (red arrow). (D) Follow-up T1-weighted sequence showing an oval soft-tissue mass with slightly hyperintense signal and epidural extension (red arrow), consistent with local relapse.

A 59-year-old man presented with progressive neck and upper thoracic pain, accompanied by weakness of the lower extremities. Neurologic examination revealed a sensory level localizing to T1. Magnetic resonance imaging (MRI) demonstrated a posterior extradural mass extending from C7 to T1, causing spinal cord compression (Figure 1). Bone scintigraphy identified a lytic lesion at T1 with a perilesional osteoblastic reaction and no evidence of distant disease. The patient’s medical history included hypertension, dyslipidemia, prediabetes, obesity, hepatic steatosis, benign prostatic hyperplasia, and hypothyroidism; serum lactate dehydrogenase (LDH) and calcium levels were within normal ranges at the time of evaluation.

**Figure 2:**
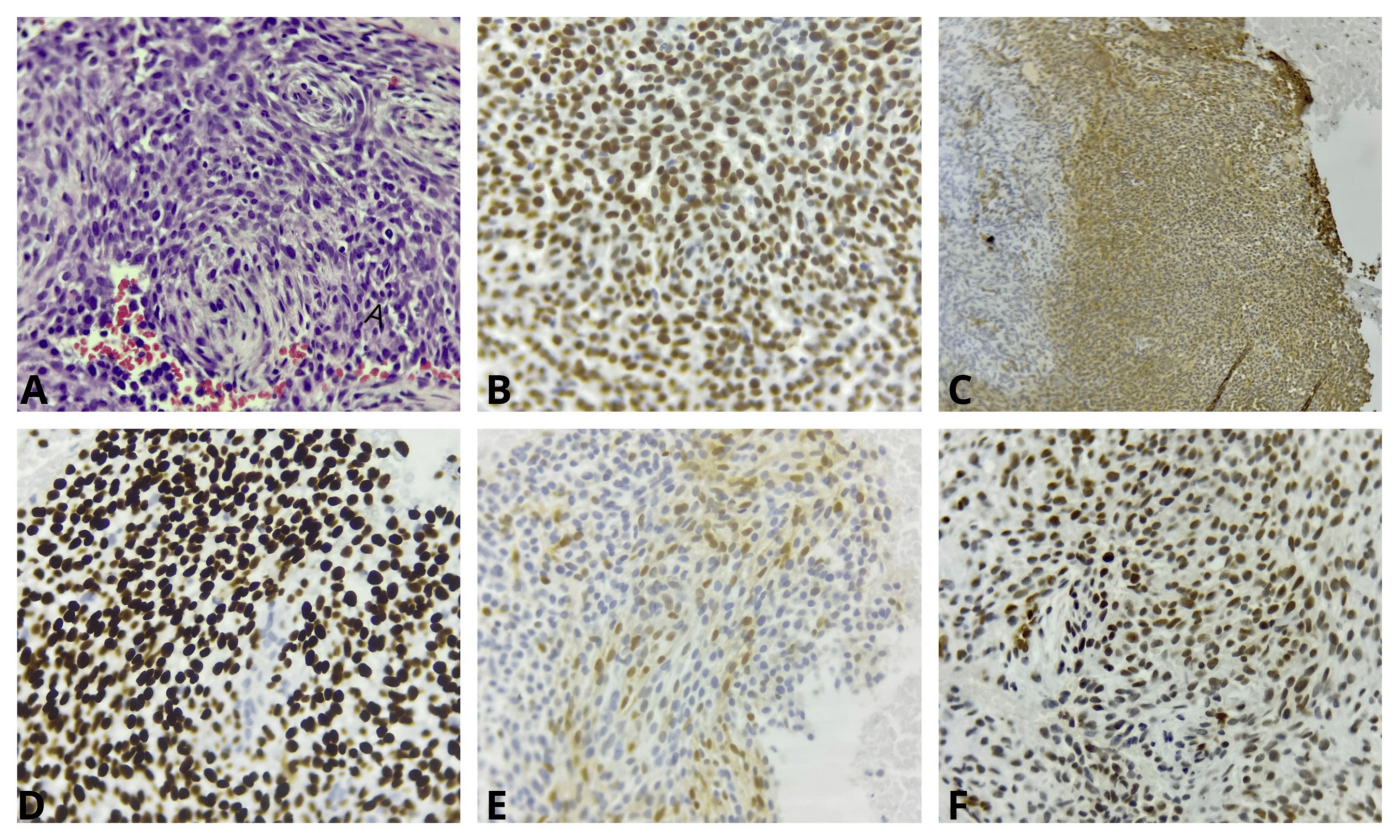
Immunohistochemical staining (A) Hematoxylin and eosin (H&E) stain showing a biphasic tumor composed of small round blue cells admixed with spindle-shaped cells, some displaying nuclear hyperchromasia. (B) Immunohistochemical staining for TLE1 showing diffuse cytoplasmic positivity. (C) CD99 demonstrating strong membranous expression. (D) SATB2 showing diffuse nuclear positivity. (E) Cyclin D1, focally positive. (F) FLI1, showing focal nuclear expression.

He received urgent radiotherapy (30 Gy in 10 fractions) for spinal cord compression, followed by posterior cervicothoracic decompression and fixation (C5-T3). Histopathologic examination revealed a spindle- and oval-cell neoplasm with hyperchromatic nuclei and nodular architecture. Immunohistochemical staining was positive for CD99, SATB2, TLE1, cyclin D1, and focal FLI1 and negative for EMA, S100, desmin, calponin, and SOX10. The Ki-67 proliferation index was 30%. Fluorescence in situ hybridization (FISH) demonstrated an EWSR1 break-apart signal pattern with amplification (3-8 copies) in approximately 70% of tumor cells, consistent with an unbalanced EWSR1 gene rearrangement. This amplification pattern is more frequently associated with non-ETS EWSR1 fusion partners. Unlike the canonical balanced translocation observed in classic Ewing sarcoma, which typically shows one fused (yellow) signal corresponding to the intact allele and one pair of separated red (5′) and green (3′) signals without amplification, non-ETS fusions may demonstrate signal amplification or complex rearrangement patterns. In particular, EWSR1-NFATC2 has been reported to exhibit tandem amplification of the fusion gene, resulting in multiple clustered signals on FISH analysis, whereas this finding is less commonly described in EWSR1-PATZ1 (Figure 2).

Adjuvant chemotherapy (MAI regimen: doxorubicin/ifosfamide) was administered. Ten months later, local disease progression was detected, and the first-line VAC protocol (vincristine, dactinomycin, and cyclophosphamide) was initiated. After the fifth cycle, a local recurrence involving the C7-T2 levels was documented, with involvement of the left brachial plexus. Second-line gemcitabine/docetaxel was subsequently started, achieving a partial response. At 18 months of follow-up, the disease continued to show local progression with no evidence of distant metastases.

## Discussion

EWSR1-rearranged spindle and round cell sarcomas have only recently been recognized within the spectrum of small round cell tumors. These neoplasms differ from classical Ewing sarcoma both morphologically and genetically, as well as in their epidemiologic profile, particularly regarding the age of onset [1-3].

Among the non-ETS fusions, tumors harboring EWSR1-NFATC2 rearrangements are typically found in adults. They often display a mixture of spindle and round cell components, show variable CD99 expression, and generally exhibit limited sensitivity to conventional Ewing-type chemotherapy [6-8]. In contrast, EWSR1-PATZ1 sarcomas tend to arise in the soft tissues of the chest or abdomen and may demonstrate focal neural or myogenic differentiation, which can make histologic recognition challenging [4,5].

In the present case, FISH analysis confirmed an EWSR1 rearrangement with multiple copy gains, but the fusion partner gene could not be identified. This limitation is common in settings lacking next-generation sequencing (NGS) or reverse transcription PCR (RT-PCR)-based fusion panels, making it difficult to classify the tumor precisely under the current WHO framework [9,10]. In resource-limited settings, where comprehensive molecular testing is not readily available, we propose that careful morphologic evaluation by an experienced sarcoma pathologist, combined with a selected immunohistochemical panel, represents a practical and effective diagnostic strategy. Although molecular confirmation remains the gold standard for precise classification under the current WHO framework, expert histopathologic assessment can substantially narrow the differential diagnosis and guide appropriate clinical management in low-resource environments.

The differential diagnosis of a round cell sarcoma harboring a non-ETS EWSR1 fusion is broad and includes the expanding family of undifferentiated round cell sarcomas. This group comprises (1) sarcomas with EWSR1 non-ETS rearrangements, (2) CIC-rearranged sarcomas, (3) BCOR-rearranged sarcomas, and (4) undifferentiated round cell sarcomas, not otherwise specified. As summarized in Table 1, important histologic and immunohistochemical differences may assist in distinguishing these entities. In addition, FISH findings can provide useful diagnostic clues. In EWSR1 non-ETS rearranged sarcomas, break-apart FISH demonstrates an EWSR1 rearrangement, often with unbalanced or amplified signal patterns, particularly in cases such as EWSR1-NFATC2. In contrast, CIC- and BCOR-rearranged sarcomas typically show a normal (non-rearranged) EWSR1 break-apart pattern, with two fused signals indicating an intact EWSR1 locus. Undifferentiated round cell sarcomas not otherwise specified likewise lack EWSR1 rearrangement on break-apart analysis [11].

**Table 1:** Comparative clinicopathologic and molecular features of round cell sarcoma families. Data synthesized from multiple published sources Table content is supported by comprehensive reviews and recent classification updates, including References [1,2,11]. NOS: not otherwise specified; FISH: fluorescence in situ hybridization

Feature	EWSR1–ETS (canonical Ewing sarcoma)	EWSR1–non-ETS (e.g., NFATC2 and PATZ1)	CIC-rearranged sarcoma	BCOR-rearranged sarcoma	Undifferentiated round cell sarcoma, NOS
Fusion partner	FLI1, ERG, ETV1, ETV4 (ETS family)	NFATC2, PATZ1, CREB1, ATF1, SMARCA5, others	DUX4 (most common), FOXO4, NUTM1	BCOR-CCNB3, BCOR-MAML3, BCOR ITD	No recurrent defining alteration
Typical age group	Children & young adults (10–25 yrs)	Young to middle-aged adults (20–50 yrs)	Young adults (20–40 yrs)	Children & adolescents (male predominance in BCOR-CCNB3)	Variable
Common sites	Diaphyseal long bones, pelvis, chest wall	Soft tissue > bone; paraspinal, deep soft tissue	Deep soft tissue (limbs, trunk); less often bone	Bone (especially long bones), pelvis; soft tissue	Variable
Histologic pattern	Uniform small round blue cells, scant cytoplasm	Mixed round and spindle cells; variable cellularity; sometimes myxoid areas	Round to slightly pleomorphic cells; lobulated growth; myxoid stroma; geographic necrosis	Round to spindle cells; sometimes fascicular areas; delicate vasculature	High-grade round cell tumor lacking defining features
Immunoprofile	CD99 diffuse membranous; NKX2.2+; FLI1+	CD99 variable/patchy; SATB2+; TLE1+; cyclin D1+; FLI1 often negative or focal	CD99 patchy; WT1 nuclear+; ETV4+	BCOR+, cyclin D1+; SATB2+ (variable); CD99 variable	Non-specific; CD99 may be patchy
EWSR1 break-apart FISH	Rearranged (balanced): 1 fused signal + 1 split (red/green separated), no amplification	Rearranged (often unbalanced): split signals ± amplification; may show clustered 5′ signals (tandem amplification, especially NFATC2)	Normal (two fused signals)	Normal (two fused signals)	Normal
Molecular mechanism	Balanced translocation t(11;22) or variants	Often unbalanced rearrangement; tandem amplification of fusion gene (esp. NFATC2)	CIC fusion gene	BCOR fusion or internal tandem duplication	Undefined
Clinical behavior	Approximately 20%–30% present with metastases at diagnosis, but chemo-responsive	Often present as large, locally aggressive tumors, may be less chemo-responsive	Aggressive, high metastatic potential	Intermediate to aggressive	Variable, often aggressive

From a therapeutic standpoint, clinical behavior appears to depend on the fusion partner. EWSR1-NFATC2 tumors are typically refractory to Ewing-type chemotherapy and radiotherapy, showing a tendency for local recurrence and indolent progression [6-8]. Conversely, EWSR1-PATZ1 sarcomas may show transient responses to doxorubicin/ifosfamide-based regimens, and anecdotal reports suggest possible benefit from agents such as pazopanib or trabectedin [4,5,9].

Because the fusion type could not be identified in our patient, Ewing-based chemotherapy was selected as the initial regimen. This approach reflects the real-world management challenges faced in resource-limited settings, underscoring the need for broader access to molecular diagnostic testing, which can significantly influence both prognostic assessment and therapeutic decision-making [10]. As molecular characterization becomes increasingly integrated into sarcoma diagnostics, accurate subclassification of undifferentiated round cell sarcomas will be essential to improve prognostic stratification and to develop subtype-specific therapeutic approaches. In summary, the case highlights the importance of recognizing EWSR1-rearranged sarcomas as distinct entities, necessitating tailored treatment strategies due to their unique molecular characteristics and clinical behavior.

## Conclusions

Diagnosing these tumors remains challenging. EWSR1-rearranged spindle and round cell sarcomas with non-ETS fusions can mimic a variety of other neoplasms, and in many cases, the available ancillary studies are insufficient for definitive classification. In the present case, diagnosis and treatment decisions relied primarily on morphologic evaluation, immunohistochemical profiling, and a basic FISH assay confirming the EWSR1 rearrangement. Despite multimodal therapy-including surgery, radiotherapy, and Ewing-type chemotherapy-the disease recurred locally, underscoring its limited responsiveness compared with conventional Ewing sarcoma. At present, management must be individualized, and broader access to advanced molecular testing would greatly enhance the precision of diagnosis and therapeutic decision-making in these rare entities. The complexity of EWSR1-rearranged sarcomas necessitates ongoing research to elucidate their distinct clinical behaviors and optimize treatment strategies tailored to individual molecular profiles.
